# Self-consistency in Bicultural Persons: Dialectical Self-beliefs Mediate the Relation between Identity Integration and Self-consistency

**DOI:** 10.3389/fpsyg.2017.00321

**Published:** 2017-03-07

**Authors:** Rui Zhang, Kimberly A. Noels, Richard N. Lalonde, S. J. Salas

**Affiliations:** ^1^Department of Psychology, Dickinson College, CarlislePA, USA; ^2^Department of Psychology, University of Alberta, EdmontonAB, Canada; ^3^Department of Psychology, York University, TorontoON, Canada; ^4^Department of Theology, Wilfrid Laurier University, WaterlooON, Canada

**Keywords:** biculturalism, self-concept consistency, bicultural identity integration, naïve dialecticism, East Asian, Latino

## Abstract

Prior research differentiates dialectical (e.g., East Asian) from non-dialectical cultures (e.g., North American and Latino) and attributes cultural differences in self-concept consistency to naïve dialecticism. In this research, we explored the effects of managing two cultural identities on consistency within the bicultural self-concept via the role of dialectical beliefs. Because the challenge of integrating more than one culture within the self is common to biculturals of various heritage backgrounds, the effects of bicultural identity integration should not depend on whether the heritage culture is dialectical or not. In four studies across diverse groups of bicultural Canadians, we showed that having an integrated bicultural identity was associated with being more consistent across roles (Studies 1–3) and making less ambiguous self-evaluations (Study 4). Furthermore, dialectical self-beliefs mediated the effect of bicultural identity integration on self-consistency (Studies 2–4). Finally, Latino biculturals reported being more consistent across roles than did East Asian biculturals (Study 2), revealing the ethnic heritage difference between the two groups. We conclude that both the content of heritage culture and the process of integrating cultural identities influence the extent of self-consistency among biculturals. Thus, consistency within the bicultural self-concept can be understood, in part, to be a unique psychological product of bicultural experience.

## Introduction

Hundreds of millions of people worldwide now could be considered *de facto* bicultural (United Nations Department of Economic and Social Affairs, 2013). Broadly speaking, biculturals are people with non-trivial exposure to and participation in at least one culture, including but not restricted to immigrants, ethnic minorities, multiracial individuals, and sojourners (Nguyen and Benet-Martínez, 2007). There are many shades of biculturalism as culture itself comes in a variety of forms (ethnicity, gender, social class, religion, etc.), but most research thus far has focused on immigrants and ethnic minorities (e.g., Asian Americans) or people who are influenced by foreign cultures (e.g., Hong Kong Chinese). Because different cultures tend to contain relatively distinct sets of norms and ideals, a unique challenge befalling biculturals is how to manage the identities associated with their cultures within themselves. Given the profound influence of culture on the self (Markus and Kitayama, 1991), it stands to reason that relating to two cultures should also shape one’s sense of self. Thus, focusing on people who are bicultural in the present article, we seek to understand the influence of managing two cultural identities on self-concept consistency, which refers to stable and non-contradictory thoughts and feelings about the self. Rooted in biculturalism research, our guiding framework regards the processes of negotiating cultural identities as being integral to being bicultural (Benet-Martínez and Haritatos, 2005; Tadmor and Tetlock, 2006; No et al., 2011; West et al., under review). In what follows, we link one specific process – individual differences in experiencing cultural identities as more or less integrated within the self – with self-concept consistency. We will show that a consistent self-concept can be understood, in part, as the unique psychological product of an integrated bicultural identity. Importantly, we argue that the experience of having an integrated bicultural identity should affect self-consistency to a similar extent across bicultural people of various ethnic heritages that nonetheless differ in terms of emphasis on the need to maintain self-consistency.

### Bicultural Identity Integration and the Bicultural Self-concept

Living in two cultural worlds requires biculturals to manage cultural complexity both socially and psychologically. Socially, being bicultural entails developing relationships with two relatively distinct communities, thus calling for effective communication with both groups and a wider linguistic or behavioral repertoire (LaFromboise et al., 1993; Noels, 2013). Psychologically, being bicultural creates accountability pressures to more than one cultural audience (Tadmor and Tetlock, 2006). The existence of different or even colliding cultural norms and modes of communication may turn inward, resulting in the need to reconcile cultures within biculturals themselves. The construct of bicultural identity integration was developed to capture individual differences among biculturals in their subjective experience of relating to both cultures (Benet-Martínez and Haritatos, 2005). Specifically, biculturals vary in how their bicultural identity is organized within the self-concept: some experience their cultural identities as harmonious or blended, whereas others experience their cultures as oppositional or compartmentalized (Benet-Martínez and Haritatos, 2005; Huynh et al., 2011). This line of research has provided extensive evidence clarifying the dimensionality, social antecedents, and psychological consequences of having a more or less integrated bicultural identity (for a recent review, see Cheng et al., 2014).

Much of the research on the bicultural self-concept has been approached from a cultural frame switching perspective. Cultural frame switching is the experience of responding to cues of cultural identity (e.g., language, cultural icons) and applying appropriate cultural knowledge and behaviors (Hong et al., 2000; also see, Chiou and Mercado, 2016). To the extent that different cultures foster contrasting senses of self (e.g., introversion vs. extraversion, Chen and Bond, 2010; self-enhancement vs. self-effacement, Lee et al., 2010), biculturals can, in principle, switch between these different self-aspects. In a similar vein, biculturals also shift their momentary feelings of cultural identification and their situated cultural identities are typically attuned to whichever culture is salient in the immediate social situation (Clément and Noels, 1992; Zhang and Noels, 2013; Noels and Clément, 2015). Research on these fronts suggest that because biculturals may be particularly prone to showing context-dependent self-views, they might be less consistent in their overall self-concept. As will be detailed below, there is a longstanding tradition in examining the ways in which the self maintains consistency in personality psychology and more recent research has shifted its focus to investigating cultural influence on self-concept consistency. Despite the common interest, however, there has been surprisingly little theoretical or empirical intersection between the two research traditions. There is virtually no research examining self-consistency among biculturals; nor is there research linking bicultural identity integration specifically to self-consistency. The present article aims to fill in this important knowledge gap.

### Self-concept Consistency and Naïve Dialecticism

Self-concept consistency or consistency within the self has figured prominently in influential perspectives on self and identity (e.g., Festinger, 1957; Block, 1961). Ever since the seminal treatises on the cultural grounding of the self (Markus and Kitayama, 1991; Shweder, 1991), however, extensive research has shown that culture moderates the importance of self-consistency. One well-studied form of self-consistency is consistency across situations or roles, the sense that one possesses a stable set of self-defining attributes that transcends context. Relative to North Americans and West Europeans, East Asians describe themselves more differently in the presence of different people (Kanagawa et al., 2001) and report more differentiation between their general self and their role-specific selves (e.g., self as a close friend or a son/daughter; Suh, 2002; Church et al., 2008). Another form of self-consistency is internal consistency of the self, which is the perception that one’s global sense of self is internally coherent and non-contradictory. East Asians are also more likely to harbor contradictory self-views or ambivalent self-evaluations simultaneously (e.g., being both extraverted and introverted; Choi and Choi, 2002; Spencer-Rodgers et al., 2004, 2009).

Not only does the cultural psychological perspective reveal cultural differences in self-consistency, it has also contributed to understanding their specific causes. A substantial amount of research indicates that cultural variations in self-consistency reflect variations in the general tendency to expect changes and tolerate contradiction in the world, which has been termed naïve dialecticism (for a review, see Spencer-Rodgers et al., 2010). Naïve dialecticism is a lay belief system that originated in East Asian (e.g., Chinese, Japanese, and Korean) philosophical traditions and consists of three core principles: (1) what is constant in life is change, (2) everything contains within itself its opposite, and (3) everything in the universe is intricately interconnected (Peng and Nisbett, 1999). In terms of acquiring knowledge, the East Asian dialectical approach questions the existence of invariant and absolute truths. This contrasts sharply with the synthesis orientation in the Western intellectual traditions that prioritizes the pursuit of unchanging truths that transcend opposing propositions (Peng and Nisbett, 1999).

Several lines of evidence support the role of naïve dialecticism as a proximal mechanism that explains cultural differences in consistency-related cognitions and emotions, including self-concept consistency that is of interest here. First, multi-national comparisons have shown that cross-national differences in cross-role consistency could not be accounted for by individualism-collectivism, but by naïve dialecticism (Church et al., 2008). This is because dialectical thinking appears to be unique to East Asian cultures and people from non-dialectical, collectivistic cultures (e.g., Mexico) are no less consistent than those from non-dialectical, individualistic cultures (e.g., Australia; for related evidence, see Schimmack et al., 2002; Scollon et al., 2005). Second, cross-national differences in self-consistency were replicated among ethnic groups within the United States. Asian Americans showed greater inconsistency across roles (English and Chen, 2007) than did European Americans. Asian Americans also showed more ambivalent (hence less consistent) global self-evaluations relative to European, Latino, and African Americans (Spencer-Rodgers et al., 2004). Last, direct evidence for the causal role of dialectical thinking in self-consistency can be seen from studies showing that cultural differences were mediated by dialectical self-beliefs (English and Chen, 2007; Boucher, 2011) and from experimental studies where dialectical thinking was manipulated (Spencer-Rodgers et al., 2004). Therefore, naïve dialecticism provides the most parsimonious explanation for cultural variations in self-consistency.

### The Present Research

The overarching goal of this article is to integrate key insights from research on both biculturalism and naïve dialecticism in the study of two forms of self-consistency among biculturals: consistency across roles and global consistency. Prior research on self-consistency with bicultural samples focused on the influence of heritage culture (Spencer-Rodgers et al., 2004; English and Chen, 2007), while paying scant attention to the possible effect of their unique bicultural experience. Biculturals may exhibit psychological tendencies that cannot be understood solely by examining their heritage cultures; these tendencies can also be understood by examining the processes that biculturals use to negotiate their cultural identities (Tadmor et al., 2012; Saad et al., 2013; West et al., under review). As such, the degree of self-consistency among biculturals may be driven by both how dialectical their heritage cultures are and how they negotiate their cultural identities.

Focusing on bicultural identity integration, our first hypothesis states that having an integrated bicultural identity would be associated with being more consistent across roles and having a more consistent global self-concept (H1). We formulated H1 for the following reasons. First, some prior work provides indirect supportive evidence. In studies of Latino American biculturals, identity integration was associated with a stronger perceived overlap between their personality and both typical Latino and American personality profiles (Miramontez et al., 2008). This suggests that by perceiving themselves as broadly compatible with both cultures, more integrated biculturals achieve more coherence within their self-concept. In another study of how Chinese Americans represented their cultures, less integrated Chinese Americans were found to provide more complex cultural representations (Benet-Martínez et al., 2006). Although the results are less tied to self-concept, we can deduce that a richer differentiation of cultures might translate into a more compartmentalized and internally tensioned self-concept, hence lower self-consistency. Thus, previous studies hint at identity integration leading to more self-consistency. More importantly, we believe there is a key conceptual reason why this may be the case. To integrate cultural identities presupposes the recognition of at least the potential for cultural conflict and consequently the need to resolve contradictions that arise from belonging to two cultures. From the non-dialectical perspective, integration is the ideal solution to possessing contradictory self-aspects as it draws attention to broader similarities and the bigger picture (Amiot et al., 2007). Contrarily, the dialectical perspective encourages a mere acceptance rather than resolution of contradiction within the self or in the social world (Peng and Nisbett, 1999; for related empirical work, see Chen et al., 2013). Managing to integrate cultural identities should thus be associated with higher self-consistency.

**FIGURE 1:**
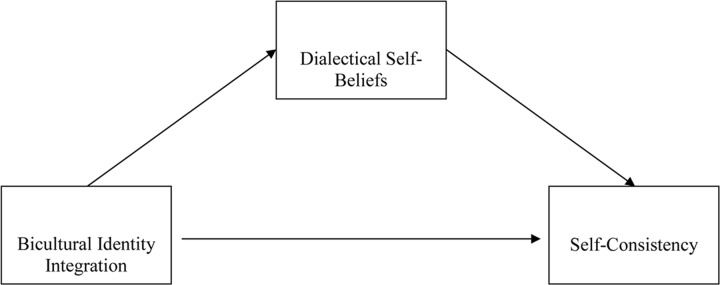
**The conceptual mediation model whereby bicultural identity integration affects self-consistency via dialectical self-beliefs**.

Our second hypothesis states that bicultural identity integration would affect cross-role and global self-consistency indirectly through dialectical self-beliefs (H2). **Figure 1** displays the conceptual mediation model. Based on the review of the literature provided earlier, dialecticism has been linked with both self-consistency and bicultural identity integration. On the one hand, given the evidence that dialecticism explains cultural variations in self-consistency, dialecticism can be conceived as a sufficient condition for reliably producing self-inconsistency. In the only published studies examining the effect of dialecticism on self-consistency among biculturals, dialecticism was found to predict variations in personality across contexts involving the use of different languages (Chen et al., 2014). On the other hand, because integration is an effective way of reducing contradiction (Peng and Nisbett, 1999), success in integrating cultural identities should reduce the propensity of using dialectical thinking. On the flip side, having difficulty with reconciling cultural identities could increase the general tendency to expect and even tolerate contradiction. In support of this, negative associations between these two variables were found in biculturals in Hong Kong and China (Chen et al., 2013). Therefore, bicultural identity integration was hypothesized to negatively predict dialectical thinking, which in turn would negatively predict self-consistency. It is worth mentioning that because a bicultural person’s level of dialectical thinking is clearly influenced by their heritage culture, our mediation model only specifies bicultural identity integration as one of the factors affecting dialectical thinking. It is also important to note that given the correlational nature of the studies we drew upon (Chen et al., 2013), it may well be that identity integration is the result, as opposed to the cause, of dialectical thinking. We will return to the issue of directionality of the mediation model in the section “General Discussion.”

Our third and final hypothesis concerns sharpening the distinction between our predictions regarding the influence of bicultural identity integration on self-consistency and the previously documented ethnic differences among bicultural groups in self-consistency. As mentioned above, different bicultural groups differ in self-consistency because their heritage cultures differ in the extent of socializing naïve dialecticism. Thus, Asian Americans tend to have a more ambivalent self-concept relative to Latino Americans (Spencer-Rodgers et al., 2004). Given our focus on common bicultural experience, we think bicultural identity integration should affect self-consistency to a similar degree across a variety of bicultural groups. For instance, both more integrated Asian biculturals and more integrated Latino biculturals should show higher self-consistency. However, this effect of bicultural identity integration is analytically distinct from the effect of heritage culture. While more integrated Asian and Latino biculturals should show more self-consistency, this need not eliminate the average self-consistency difference between the two groups; this difference is driven largely by ethnic difference in the dialectical vs. non-dialectical tradition. Hence, our third hypothesis is as follows: biculturals of dialectical ethnic heritage would show lower self-consistency relative to those of non-dialectical ethnic heritage (H3). This hypothesis represents a conceptual replication of previous findings.

In the following, we present four studies designed to test the three hypotheses (Study 1: H1; Study 2: H1, H2, and H3; Study 3: H1 and H2; Study 4: H1 and H2). In all studies, we defined biculturals in terms of cultural exposure. By this definition, biculturals are those with involvement in two cultures (e.g., ethnic minorities), regardless of their subjective identification with them (Nguyen and Benet-Martínez, 2007). Such a demographically based definition allows us to maximize the number of bicultural participants that could be included in each study. Another issue is the dimensionality of bicultural identity integration. We followed the widely adopted distinction between the cognitive and the affective components of integration (Huynh et al., 2011). While the cognitive dimension captures whether the bicultural identity is represented jointly or separately in the self, the affective dimension reflects if the bicultural identity feels congruent or tensioned. The two dimensions are both conceptually and empirically distinct (Huynh et al., 2011). In all studies, we assessed both dimensions and explored whether there would be any difference in terms of their associations with self-consistency.

## Study 1

We conducted our first study to seek preliminary support for the hypothesized association between bicultural identity integration and consistency across roles (H1). We recruited a Chinese Canadian sample and assessed the two dimensions pertaining to their bicultural identity integration (Benet-Martínez and Haritatos, 2005) and how similarly they rated their personality in the context of close friendship that typically involves two groups of cultural audiences (Tadmor and Tetlock, 2006).

Ethical approval for all the studies presented here was obtained from the following ethics board: Research Ethics Office at the University of Alberta (Studies 1–3) and Office of Research Ethics at York University (Study 4). In all studies, participants gave informed consent by signing or checking a box next to the line that said “I consent.”

### Method

#### Participants

Participants were 81 Chinese Canadians from introductory psychology classes (70% women, *M*_age_ = 19.23, *SD*_age_ = 1.71). As citizens or permanent residents, about half (54%) of them were born in Canada; the mean length of residence among those foreign-born was 10.63 years (*SD* = 3.94)^1^.

#### Measures

In small groups, participants arrived at a computer lab and completed an online survey that assessed bicultural identity integration, self-concept consistency across roles, and demographic questions. They also completed other measures developed for a different study reported elsewhere (Zhang et al., 2016, Study 1). More details regarding the measures appear below.

##### Bicultural identity integration

Participants completed the Bicultural Identity Orientation Scale (BIOS; Comanaru et al., under review) by rating their agreement with each statement on a 6-point scale (1 = *strongly disagree*, 6 = *strongly agree*). We focused on the two subscales most germane conceptually to identity integration: the extent to which one feels tension between their two cultures (conflict, four items; e.g., “I have difficulty reconciling the differences between my ethnic culture and the Canadian culture”; α = 0.70), and the extent to which one perceives oneself to be a blended product of two cultures (hybridity, four items; e.g., “I feel my identity is a hybrid of two cultures”; α = 0.79). The lower the conflict scores, the higher the hybridity scores and the more integrated one’s bicultural identity is.

##### Consistency across roles

We adopted the Trait-Role Questionnaire that has been widely used in cross-cultural research investigating self-concept consistency (e.g., Church et al., 2008). It consisted of 40 trait adjectives tapping the Big Five dimensions (Extraversion, Agreeableness, Conscientiousness, Emotional Stability, and Openness to Experience), with each dimension indexed by eight items (see Church et al., 2008). Using a 7-point scale ranging from *very uncharacteristic of me* to *very characteristic of me*, participants then rated the extent to which the same 40 Big Five traits described them while being with a close friend who is European Canadian and while being with a close friend who is Chinese Canadian. That is, they provided contextualized personality ratings in two friendship contexts. To facilitate that, they were first asked to write down the initials of each friend and think about their relationship with the person. If they could not think of a close friend, they were instructed to write down the initials of a casual friend instead. The order of the friendship contexts and the traits within each friendship was randomized for each participant.

We operationalized self-concept consistency in terms of profile similarity, that is, the extent to which an individual’s personality profile in one role is similar to his or her personality profile in another role. In our case, we computed within-participant correlations between the 40 self-with-Chinese-Canadian-friend personality ratings and the 40 self-with-European-Canadian-friend personality ratings. A larger correlation between the two personality profiles indicates being more consistent across roles.

### Results and Discussion

**Table 1** lists the descriptive statistics for all the variables. As predicted, having a more integrated bicultural identity was associated with being more consistent across roles. Specifically, after the *r*-to-*Z* Fisher transformation was performed on cross-role consistency, it was correlated positively with hybridity (*r* = 0.31, *p* = 0.005) and negatively with conflict, albeit non-significantly (*r* = -0.18, *p* = 0.10). Experiencing cultural identities as discrepant with each other was more strongly associated with the perception that one acts differently with friends associated with each culture. Thus, Chinese Canadians’ internal representation of cultures appears to be manifested outwardly in the form of personality perception in the close friendship domain. It was also observed that thinking rather than feeling that one’s bicultural identity is integrated – the cognitively based aspect – mattered more. Because we did not predict this finding, we sought to replicate it first in other bicultural samples.

**Table 1:** Descriptive Statistics from Study 1 to Study 3.

Variable	Chinese Canadians (Study 1)		East Asian Canadians(Study 2)		Latino Canadians(Study 2)		European Canadians(Study 2)		Bicultural Canadians(Study 3)	
					
	*M*	*SD*	*M*	*SD*	*M*	*SD*	*M*	*SD*	*M*	*SD*
Consistency across roles^a^	0.61	0.27	0.47	0.27	0.64	0.24	0.61	0.25	0.49	0.28
Bicultural identity integration										
Hybridity	4.58	0.86	4.63	0.91	4.55	1.04			4.68	1.09
Conflict	2.28	0.85	2.40	0.90	2.48	1.18			2.17	1.04
DSS overall			4.01	0.44	3.63	0.63	3.62	0.55	3.80	0.54
Contradiction subscale			4.40	0.51	4.07	0.79	4.02	0.73	4.21	0.65
Cognitive subscale			3.60	0.71	3.22	0.87	3.23	0.79	3.40	0.77
Behavioral subscale			3.84	0.71	3.39	0.80	3.40	0.72	3.58	0.69


*DSS, dialectical self scale. ^a^Values in this row represent correlation coefficients.*

## Study 2

Study 2 was designed to extend it in a number of ways. First, given that people in East Asian cultural contexts tend to be more dialectical, it is unclear whether the finding was unique to only biculturals of dialectical heritage background. Thus, in addition to an East Asian bicultural sample, we recruited a Latino bicultural sample as people in Latin American cultural contexts have been shown to be collectivistic but are equally synthesis-oriented as people in European American or Canadian cultural contexts. We expected bicultural identity integration to be associated with consistency across roles with each bicultural sample. Second, we broadened the friendship context assessed in the consistency measure to encompass another common interpersonal context: interactions with one’s parents. That is, we examined consistency between self-as-a-friend and self-as-a-son/daughter. Third, we also assessed dialectical beliefs to test H2 that identity integration affects consistency across roles indirectly via dialectical self-beliefs. Finally, we tested H3 that ethnic differences in self-concept consistency between the two bicultural samples should mirror differences between dialectical and non-dialectical cultures. As explained above, because different bicultural groups are socialized into different heritage cultures, their bicultural experiences might not eliminate between-group differences due to the influence of heritage cultures. Because previous research shows people of East Asian cultural heritage to be more dialectical and less consistent relative to people of Latino cultural heritage (Schimmack et al., 2002; Spencer-Rodgers et al., 2004; Church et al., 2008), we expected East Asian biculturals to be less consistent across roles, as a whole, than Latino biculturals. We also recruited a third sample as a prototype group of monocultural Canadians: European Canadians who were born in the country. Although not bicultural as commonly defined, European Canadians share the synthesis-oriented ethnic heritage with Latino Canadians (Schimmack et al., 2002; Spencer-Rodgers et al., 2004; Church et al., 2008). If it were true that ethnic differences in self-consistency are largely driven by dialecticism, both Latino and European Canadians should be more consistent relative to Asian Canadians. These ethnic differences, in turn, should be mediated by dialectical self-beliefs.

### Method

#### Participants

Participants were 65 European Canadians (68% women, one unspecified), 101 East Asian Canadians (55% women, one unspecified), and 70 Latino Canadians (61% women, one unspecified). Both European and East Asian samples as well as approximately half of the Latino sample consisted of undergraduates enrolled in introductory psychology courses who received partial course credit. The rest of the Latino sample consisted of students recruited via campus flyers or snowball sampling and they received $10 for participation. As a result of this recruitment strategy, the Latino participants were on average older and more variable in age (*M* = 22.16, *SD* = 5.85), compared with their European (*M* = 19.09, *SD* = 1.67) and East Asian (*M* = 18.61, *SD* = 1.65) counterparts. Hence, in the following analyses concerning group comparisons, we controlled for age. All participants were Canadian citizens or permanent residents. All European, 65% of the East Asian, and 47% of the Latino participants were born in Canada, respectively. Of those foreign-born, the mean length of residence was 9.37 years (*SD* = 4.49) and 9.32 years (*SD* = 5.01) for East Asian and Latino Canadians, respectively.

#### Measures

Upon arrival in a lab, participants filled out a survey (either paper-and-pencil or on a computer) comprised of the following measures and demographic questions.

##### Bicultural identity integration

It was assessed with the same BIOS conflict and hybridity subscales as used in Study 1 (Comanaru et al., under review). Only the two bicultural groups completed them. The αs were 0.71 (conflict) and 0.80 (hybridity) for the East Asian Canadians; 0.72 (conflict) and 0.76 (hybridity) for the Latino Canadians.

##### Consistency across roles

Using an 8-point scale, all participants rated the extent to which the same 40 Big Five traits described them in two interpersonal contexts. Because we were interested in consistency across common roles people assumed, not just roles that may have specific meaning to biculturals, we broadened the close friendship context to include being with one’s parent and a friend, the closeness of which was not defined. Again, the traits were presented in different orders for each role and the order of the roles was randomized for each participant. To index consistency across roles, we computed with-participant correlations between the two sets of the contextualized personality ratings.

##### Dialectical self-beliefs

All participants completed the 32-item Dialectical Self Scale (DSS; Spencer-Rodgers et al., 2015, Unpublished) that assesses dialectical beliefs in the domains of contradictions (e.g., “When I hear two sides of an argument, I often agree with both”), cognitive change (e.g., “I often find that my beliefs and attitudes will change under different contexts”), and behavioral change (e.g., “I often change the way I am, depending on who I am with”). Responses were recorded on a 7-point scale (1 = *strongly disagree*, 7 = *strongly agree*). The αs for the overall DSS ranged from 0.72 to 0.80. Because previous work has used either a specific subscale or the overall scale to explain cultural differences in self-concept consistency (English and Chen, 2007; Church et al., 2012), we explored the effects of both overall DSS and its subscales.

### Results and Discussion

#### Within-Group Results

In support of H1, bicultural identity integration was correlated with consistency across roles in each bicultural group. Specifically, the *r*-to-*Z* Fisher transformed consistency index was correlated positively with hybridity (East Asian: *r* = 0.20, *p* = 0.047; Latino: *r* = 0.11, *p* = 0.38) and negatively with conflict (East Asian: *r* = -0.17, *p* = 0.10; Latino: *r* = -0.26, *p* = 0.03). Interestingly, consistency was more strongly associated with the cognitive dimension of integration for East Asian biculturals, which is in line with the Study 1 finding, whereas it was more closely associated with the affective dimension for Latino biculturals.

To test H2 concerning the mediation hypothesis, we first established if DSS would be correlated with both bicultural identity integration and consistency within each group. Among the East Asian biculturals, only behavioral DSS was correlated with hybridity (*r* = -0.25, *p* = 0.013) and consistency (*r* = -0.40, *p* < 0.001). We then proceeded to test mediation with Preacher and Hayes’ (2008) bootstrapping method. Five thousand bootstrapping resamples were performed. The bias-corrected 95% CI for the indirect effect of hybridity on consistency via the mediator of behavioral DSS did not contain zero [0.005, 0.09], providing support for H1. In a regression model, the total effect of hybridity on cross-role consistency was reduced from β = 0.20, *t* = 2.01, *p* = 0.047 to β = 0.11, *t* = 1.13, *p* = 0.26 once behavioral DSS was introduced to the model. Among the Latino biculturals, overall DSS was correlated with conflict (*r* = 0.57, *p* < 0.001), hybridity (*r* = -0.30, *p* = 0.013), and consistency (*r* = -0.44, *p* < 0.001). Given the contemporary perspective that a significant overall effect is no longer prerequisite for testing mediation (MacKinnon et al., 2002; Shrout and Bolger, 2002), we proceeded to test the indirect effect of hybridity on cross-role consistency via dialectical beliefs as well, even if the total effect was non-significant. With 5,000 bootstrapping resample, the bias-corrected 95% CI for the indirect effect of conflict on consistency via DSS did not contain zero [-0.17, -0.04]. In a regression model, the total effect of conflict on cross-role consistency diminished from β = -0.26, *t* = -2.22, *p* = 0.03 to β = -0.02, *t* = -0.13, *p* = 0.90 once overall DSS was introduced to the model. Similarly, the bias-corrected 95% CI for the indirect effect of hybridity on consistency via DSS did not contain zero [0.02, 0.12]. Both results are in line with H2.

To summarize, we replicated the overall positive association between bicultural identity integration and consistency among both bicultural groups, providing further support for H1. A more integrated bicultural identity (in the form of higher hybridity and lower conflict) was related to a more consistent sense of self between being with one’s parent and being with one’s friend. Of note, the two bicultural groups differed in which aspect of identity integration was more strongly linked with cross-role consistency. The East Asian biculturals are akin to the Chinese biculturals from Study 1 in that cross-role consistency was better predicted by how they perceived their cultural identities to be integrated, whereas how consistent Latino biculturals were across roles was more driven by their affective reactions. Furthermore, we found evidence consistent with H2 that posited dialectical beliefs as one mechanism through which identity integration influences self-consistency. Judging by differences in the strength of associations among those variables, dialectical beliefs appear to be more causally proximal to cross-role consistency relative to either hybridity or conflict. In other words, the relatively weak associations between bicultural identity integration and cross-role consistency could be an indication that the former exerts a more distal causal influence. Finally for Latino biculturals, dialectical beliefs were suggested as both a pathway from conflict to cross-role consistency and a pathway from hybridity to cross-role consistency. The latter was true despite the non-significant overall effect. The juxtaposition between a non-significant total effect and a significant indirect effect suggests that compared with the affective dimension, the cognitive dimension of identity integration might be a more distal causal agent of cross-role consistency for Latino biculturals.

#### Between-Group Results

To test H3, we first compared group differences in Fisher *r*-to-*Z* transformed consistency across roles by conducting a one-way analysis of covariance (ANCOVA) with ethnicity (European, East Asian, or Latino) as the grouping variable while controlling for age. It should be noted that results remained the same if age was not controlled for but we opted to adjust its effect because the Latino sample was older as stated before. **Table 1** lists unadjusted, raw means and standard deviations for each group. Results revealed significant group differences, *F*(2,230) = 10.51, *p* < 0.001, $\eta_{p}^{2}$ = 0.08. Post hoc pairwise comparisons were conducted with Bonferroni correction. As predicted, both European and Latino Canadians were more consistent across roles than the East Asian Canadians, *p* = 0.002, 95% CI [-0.38, -0.07]; *p* < 0.001, 95% CI [-0.45, -0.12] respectively, while the former two groups did not differ from each other, *p* = 1.0. Next, another one-way ANCOVA comparing group differences in DSS while controlling for age showed a significant effect, *F*(2,230) = 11.77, *p* < 0.001, $\eta_{p}^{2}$ = 0.09. Pairwise comparisons with Bonferroni correction were again in line with H1. East Asian Canadians endorsed dialectical beliefs more than European Canadians, *p* < 0.001, 95% CI [0.17, 0.58], and Latino Canadians, *p* = 0.001, 95% CI [0.11, 0.55]. There was no difference between the latter two, *p* = 1.0.

In view of the ethnic differences in dialectical self and self-concept consistency, we proceeded to test a mediation model whereby dialectical self was posited to account for the group differences in consistency. We examined this indirect effect with the MEDIATE bootstrapping procedure developed by Hayes and Preacher (2014), which allows for an omnibus test of mediation involving multiple groups. In the mediation model, the predictor was comprised of two dummy-coded ethnicity variables (East Asian = 0, European = 1, Latino = 0; East Asian = 0, European = 0, Latino = 1). The consistency scores were then regressed onto the dummy-coded ethnicity variables with DSS entered as the mediator and age as the covariate. DSS significantly predicted consistency, *B* = -0.27, *p* < 0.001, while the omnibus effect of ethnicity was reduced from *F*(2,230) = 10.51, *p* < 0.001 to *F*(2,229) = 4.57, *p* = 0.01. Specifically, the previously significant East Asian-European and East Asian-Latino differences became less significant, *B* = 0.13, *p* = 0.04; *B* = 0.19, *p* = 0.004, respectively. In support of mediation, the bias-corrected 95% confidence interval (CI) constructed with 5,000 resamples did not contain zero [-0.05, -0.007], suggesting a significant omnibus effect of mediation. More specifically, DSS significantly mediated both the East Asian-European difference, 95% CI [0.05, 0.17], and the East Asian-Latino, 95% CI [0.04, 0.16].

To summarize, H3 received full support. First, we replicated findings from previous research showing ethnic differences between Asian and European Americans in cross-role consistency (English and Chen, 2007). Second, the East Asian-Latino difference in cross-role consistency dovetails with other research that examined national differences between East Asian and Latino populations in similar domains (Schimmack et al., 2002; Church et al., 2008). Finally, the mediation results clarify the causal role of dialecticism in producing the ethnic differences above. All in all, while the within-group results reveal the working of bicultural experience, the between-group results confirm the imprint of cultural heritage on self-consistency. Importantly, the within-group dynamic does not seem to eliminate the between-group differences in self-consistency, suggesting identity integration and heritage enculturation as two relatively distinct processes that shape one’s level of self-consistency.

## Study 3

In Study 3, we examined the generalizability of Study 2’s key findings in an ethnically heterogeneous bicultural sample. Specifically, we aimed to further replicate (1) the association between bicultural identity integration and consistency across roles; (2) the mediating role of dialectical self-beliefs.

### Method

#### Participants

Participants were 163 Canadians of non-European descent from introductory psychology classes (58% women, one unspecified; *M*_age_ = 18.76, *SD*_age_ = 1.90). Slightly more than half (58%) identified their cultural heritage as East Asian, followed by South Asian (27%), Middle Eastern (7%), African (6%), and Latino (2%). About half (48%) were Canadian-born, with the rest reporting their mean length of residence to be 9.60 years (*SD* = 4.59). The original sample included two international students, who were dropped from the analyses reported herein.

#### Measures

In small-group settings, participants completed a survey that contained the same measures as in Study 1. The respective αs were: conflict = 0.78, hybridity = 0.82, and DSS (overall) = 0.79, DSS (contradiction) = 0.67, DSS (cognitive) = 0.70, DSS (behavioral) = 0.50.

### Results and Discussion

**Table 1** lists the descriptive statistics of all the variables. The *r*-to-*Z* Fisher transformed consistency index was correlated negatively with conflict (*r* = -0.21, *p* = 0.006), but not hybridity (*r* = 0.06, *p* = 0.47). Because only cognitive DSS was correlated with both conflict (*r* = -0.18, *p* = 0.02) and cross-role consistency (*r* = -0.19, *p* = 0.02), we tested mediation via cognitive DSS with 5,000 bootstrapping resamples. The bias-corrected 95% CI did not contain zero [-0.03, -0.0006]. The total effect of conflict was reduced from β = -0.21, *t* = -2.77, *p* = 0.006 to β = -0.19, *t* = -2.39, *p* = 0.02 once cognitive DSS was controlled for.

Those results depart somewhat from the specific findings from previous studies. Given that about half of the current sample were of East Asian heritage, that conflict but not hybridity was significantly associated with cross-role consistency contradicts what was found in both Studies 1 and 2. However, the core tenets of H1 and H2 were largely borne out. One of the identity integration dimensions was related to self-consistency, although it is less clear which dimension was more causally proximal. Furthermore, dialectical self-beliefs, especially components that are most conceptually germane (cognitive or behavioral DSS), mediated the effect of identity integration on self-consistency.

## Study 4

In the final study, we sought converging evidence for H1 and H2 by extending the previous studies in two ways. The most important change was to shift attention to another aspect of self-concept consistency: global consistency. A consistent sense of self entails not only a core set of self attributes that is stable from situation to situation but also an overall perception that one possesses coherent self-knowledge and unambiguous feelings toward the self. The latter is often understood as internal consistency of the self. By extension, a dialectical self-concept should manifest itself in the form of exhibiting internally inconsistent judgment about the self (Choi and Choi, 2002; Hamamura et al., 2008; Spencer-Rodgers et al., 2009). Thus in this study, we tested H1 and H2 with measures tapping global self-consistency, which we operationalized as maintaining a consistent judgment of one’s general personality and a non-contradictory self-evaluation. Second, because the majority of our bicultural sample in Study 3 were of Asian descent, we recruited another bicultural sample from the Toronto metropolitan area to further increase ethnic diversity. Toronto is home to the largest number of immigrants and ethnic minorities, making it the most ethnically diverse urban area in Canada. For example, ethnic minorities account for about 47% of Toronto’s total population (Statistics Canada, 2011).

### Method

#### Participants

Our sample included 186 introductory psychology students of non-European descent at a Canadian university in Toronto (76% women, one unspecified; *M*_age_ = 21.15, *SD*_age_ = 3.73). As such, cultural heritages of this sample were more heterogeneous than previous studies: 37% South Asian, 20% Middle Eastern, 17% Black, 10% East Asian, 7% bi- or multi-racial, 5% Latino, and 4% other. While less than half (42%) were Canadian-born, those who were foreign-born had spent an average of 10.75 years (*SD* = 5.44) in Canada. Because the participants completed the survey online, we interspersed it with attention checks that instructed participants to respond in a particular way (Marjanovic et al., 2015). Inattentive participants were defined as those who responded incorrectly on at least two thirds of those instructional items (Marjanovic et al., 2015). Our original sample consisted of 34 more participants who, judging by this criterion, needed to be excluded.

#### Measures^2^

Participants completed an anonymous survey online that included the following measures and demographic questions. The order of items within each measure was randomized for each participant.

##### Bicultural identity integration

It was assessed with the 20-item Bicultural Identity Integration Scale-Version 2, which was developed by Huynh et al. (2011; BIIS-2). BIIS-2 assesses two components of bicultural identity integration: blendedness (e.g., “I relate better to a combined culture than to my heritage or Canadian culture alone”), which is conceptually analogous to hybridity; harmony (e.g., “I feel torn between my heritage and Canadian cultures”, reverse-coded), which corresponds with the opposite of conflict (see Zhang et al., 2014). Participants rated each item on a 5-point scale (1 = *strongly disagree*, 5 = *strongly agree*) and we averaged the respective items after reverse-scoring the appropriate ones to form subscale scores (blendedness: α = 0.70; harmony: α = 0.89). The higher the blendedness and harmony scores, the more integrated one’s bicultural identity is.

##### Dialectical self-beliefs

Participants completed the same DSS on a 7-point scale. Although the overall α was acceptable (0.81), the αs for each subscale varied: contradiction (0.56), cognitive (0.72), and behavioral (0.51).

##### Global self-consistency

We measured global self in two domains: personality and self-evaluation. First, participants indicated how self-descriptive each of 28 personality traits was on a 7-point scale, ranging from 1 (*not at all like me*) to 7 (*very much like me*). To assess the extent to which biculturals possess contradictory traits, we followed Spencer-Rodgers et al. (2007, Study 2) in selecting half of the traits to be conceptually contradictory with the other half. As a result, there were 14 pairs (e.g., quiet/talkative, humble/proud, shy/sociable). Second, participants completed the Rosenberg Self-Esteem scale (Rosenberg, 1965) on a 7-point scale that ranged from 1 (*not at all*) to 7 (*very much*). Given our interest in evaluative contradiction, we examined the positive and negative self-evaluations separately. Thus, positive self-esteem was computed as the mean of the positive items, while negative self-esteem the mean of the negative items (see Spencer-Rodgers et al., 2004, Study 1).

To compute global self-consistency in personality judgment and self-evaluation, we adopted three indices that were developed initially in the attitudinal ambivalence literature but have been incorporated into research on the influence of dialecticism (e.g., Spencer-Rodgers et al., 2004; Boucher, 2011). Specifically, we used Similarity Intensity Model (SIM; formula: 3*S* -*L*, with *S* being the smaller value and L being the larger value), the Conflicting Reactions Model (CRM; formula: 2 × *S*), and the Gradual Threshold Model (GTM; formula: 5*S*^0.50^ -*L*^1/S^). Although the three formulae differ in how much weight is given to the numerically smaller response in comparison to the numerically larger response, all capture the degree to which one responds in a contradictory or inconsistent way (Spencer-Rodgers et al., 2004). For instance, someone who answered “7” and “7” on a pair of contradictory items will receive the highest inconsistency score for all three indices, whereas someone who answers “1” and “7” or “7” and “1” will be assigned the lowest inconsistency score for all three. Someone rating oneself as “4” and “4” will receive an intermediate inconsistency score. For consistency in personality judgment, we applied the given formula to obtain a single value for each pair of contrasting traits and then averaged across all the 14 values to form one mean consistency index for each participant. For consistency in self-evaluation, we took the mean of the positive and negative self-evaluations, respectively, before they were entered in the given formula. This yielded a single consistency index for each participant as well. For both consistency indices, a higher score indicates being more globally inconsistent.

### Results and Discussion

To test H1, we correlated harmony and blendedness with the global consistency indices. As displayed in **Table 2**, the associations were in the predicted direction, but only four of them reached statistical significance and they all involved self-evaluative consistency. Thus, a more integrated bicultural identity (i.e., higher harmony and blendedness) was related to global consistency in self-evaluations, but not global consistency in personality judgment.

**Table 2:** Intercorrelations among BII, global consistency in personality, and global consistency in self-evaluation (Study 4).

Variable	*M*	*SD*	1	2	3	4	5	6	7	8
(1) BII-harmony	3.52	0.80	–							
(2) BH-blendedness	3.67	0.59	0.48***	-						
(3) SIM (personality)	4.40	1.77	-0.08	-0.14	–					
(4) CRM (personality)	6.66	1.22	-0.05	-0.09	0.95***	-				
(5) GTM (personality)	6.56	1.49	-0.07	-0.11	0.94***	0.88^∗∗∗^	-			
(6) SIM (self-evaluation)	3.36	4.07	-0.18*	-0.15^∗^	0.14	0.09	0.16^∗^	-		
(7) CRM (self-evaluation)	5.94	2.42	-0.17*	-0.12	0.15*	0.07	0.17^∗^	0.98^∗∗∗^	-	
(8) GTM (self-evaluation)	6.01	3.28	-0.17*	-0.13	0.14	0.10	0.16^∗^	0.95^∗∗∗^	0.94^∗∗∗^	-


*BII, bicultural identity integration; SIM, similarity intensity model; CRM, conflicting reactions model; GTM, gradual threshold model. ^∗^*p* < 0.05, ^∗∗∗^*p* < 0.001.*

As discussed in Study 2, a mediation test can be conducted, even if the total effect was non-significant (MacKinnon et al., 2002; Shrout and Bolger, 2002). We thus proceeded to test H2. We first established that overall DSS was indeed correlated with harmony (*r* = -0.26, *p* < 0.001) and all the global consistency indices (*r*s ranging from 0.24 to 0.42, *p*s < 0.01). Separate mediation analyses with 5,000 bootstrapping resamples showed that none of the bias-corrected 95% CIs for the indirect effect of harmony on global consistency via overall DSS contained zero (see **Table 3**). Furthermore, behavioral DSS was found to correlate significantly with both blendedness (*r* = -0.15, *p* = 0.04) and the global consistency indices (*r*s ranging from 0.24 to 0.41, *p*s < 0.01). Additional mediation analyses similarly demonstrated that blendedness affected global consistency via behavioral DSS (see **Table 3**). Despite the weak or non-significant total effects of harmony and blendedness, the indirect effects via dialectical beliefs were robust. Such discrepancy in strength between the overall and indirect effects again suggests that the presumed causal effect of dialectical beliefs might be more proximal, whereas identity integration represents the more distal end of the causal process. Thus, one potential explanation for the non-significant total effects on global consistency in personality judgment might be that the causal effect of identity integration is more distal and thus smaller in magnitude than expected. We will return to this issue in the section “General Discussion” to consider other possibilities.

**Table 3:** Indirect effects of bicultural identity integration on global consistency in personality and self-evaluation through dialectical self (Study 4).

	BII-Harmony		BII-Blendedness	
		
Global consistency indices	Indirect effect via DSS	95% CI	Indirect effect via behavioral DSS	95% CI
Personality				
SIM	-0.19	[-0.34, -0.08]	-0.13	[-0.35, -0.01]
CRM	-0.10	[-0.21, -0.04]	-0.07	[-0.22, -0.005]
GTM	-0.18	[-0.34, -0.08]	-0.12	[-0.31, -0.004]
Self-evaluation				
SIM	-0.56	[-1.06, -0.22]	-0.42	[-1.00, -0.02]
CRM	-0.33	[-0.62, -0.13]	-0.25	[-0.56, -0.005]
GTM	-0.42	[-0.87, -0.15]	-0.32	[-0.75, -0.0001]


*BII, bicultural identity integration; DSS, dialectical self scale; SIM, similarity intensity model; CRM, conflicting reactions model; GTM, gradual threshold model.*

## General Discussion

In the present research, we combine key insights from research on biculturalism and naïve dialecticism to understand self-concept consistency among biculturals. Across four studies, we examined whether and how individual differences in bicultural identity integration is associated with self-concept consistency. In line with H1, experiencing cultural identities as being integrated into a coherent whole was associated with enacting a similar personality profile in different roles (Studies 1–3). It also related to making unambiguous evaluations of one’s global self (Study 4). Despite ethnic variations within, bicultural Canadians are similarly shaped by the way in which they negotiate their cultural identities. In other words, this effect does not seem to depend on the types of culture biculturals inherit or whether both cultures are congruent with each other in dialectical traditions. It should apply to biculturals with both non-dialectical cultures (e.g., Latino Canadians), biculturals with one dialectical culture and one non-dialectical culture (e.g., East Asian Canadians or European Canadians living in East Asia), and presumably those with both dialectical cultures (e.g., Japanese of Chinese heritage). Next, our H2 regarding the indirect effect of dialectical self-beliefs received support (Studies 2–4). As cultural identities are better integrated within the self, biculturals tend to endorse dialectical beliefs less, which is in turn associated with being more consistent with regard to role enactment and one’s general sense of self. This set of mediation evidence adds to the usefulness of naïve dialecticism as an explanatory tool for understanding national and ethnic differences in a wide array of consistency-related phenomena (Spencer-Rodgers et al., 2010). Last, we replicated the differences between bicultural groups of dialectical and non-dialectical ethnic heritages that were found in prior research (H3). Latino Canadians (and European Canadians) showed, on average, higher consistency across roles than did East Asian Canadians and such difference was mediated by dialectical self-beliefs (Study 2). Thus, it appears that biculturals have at least two relatively independent sources that influence their levels of self-consistency: their bicultural experience as well as their heritage culture.

Despite the overall evidence, however, there were some questions as to the magnitude and consistency of effects. A case in point is the magnitude of the overall effects of identity integration in Study 4: the overall effects of harmony and blendedness on global self-consistency were either non-significant or weak. As mentioned above, it may be that those direct effects represent rather distal effects, which are difficult to detect in samples that are not particularly large in size. This would suggest the causal impact of identity integration may be temporally removed such that it would take quite some time for it to spill over to other life domains. Thus, its subtle, cumulative effects may be impractical to capture in cross-sectional studies, especially among emerging adults (cf. Tadmor et al., 2012). Moreover, a related possibility is that there exist factors that moderate the effects of identity integration on self-consistency. One such moderator may be the operationalization of biculturalism. We used a broad criterion of inclusion (i.e., exposure-based definition) in all studies, but focusing on biculturals who are oriented to both cultures (i.e., identification-based definition) may strengthen the effects of identity integration as both cultures would presumably be central to the sense of self. From a socio-cognitive perspective (Tadmor and Tetlock, 2006; Amiot et al., 2007), there would be little incentive to integrating group identities within the self if one does not categorize oneself as being part of those groups or feel accountable to them (e.g., those who exclude one culture from self-categorization by adopting predominantly assimilation or separation acculturation strategy). Another moderator of the effects of identity integration on self-consistency may be the form of self-consistency. The first three studies differ from Study 4 not only in the measurement of identity integration but also the form of self-consistency assessed. Thus, it is not clear if the weaker effects of identity integration in Study 4 are attributable to either difference or both. To confirm that the effects of identity integration are stronger on cross-role than global self-consistency, for instance, both forms of self-consistency will need to be assessed in the same study in the future. Future research may also benefit from using more spontaneous and implicit measures of global self-consistency (e.g., Spencer-Rodgers et al., 2009).

With respect to the consistency of effects, our studies varied in which dimension (cognitive vs. affective) of identity integration was more strongly linked with self-consistency in different bicultural groups. We offer three conceptually distinct explanations. To begin with, with more diverse bicultural groups (Studies 2 and 3), the affective dimension seemed to be a stronger predictor of self-consistency. This may reflect the general principle that negative affect powerfully shapes psychological experiences (Baumeister et al., 2001) or more specifically that reducing or avoiding conflict is the primary motive that guides other identity strategies (Huynh et al., 2011; Hirsh and Kang, 2016). There also appear to be some ethnic variations. In Studies 1 and 2, the cognitive dimension was a stronger predictor than the affective dimension among East Asian biculturals, whereas the opposite was true for Latino biculturals. However, it is unclear what may underlie such ethnic difference. Finally, there may be a matching effect such that the effect is stronger when the cognitive dimension is matched with a cognitive outcome or when the affective dimension is matched with an affective outcome. So the effects of affect could have been more consistent, had cross-role consistency been assessed in the form of feeling good vs. bad across contexts. In support of this reasoning, because affect can signal identity motives (Zou et al., 2008), it is identity conflict that predicted why some biculturals reacted contrastively to cultural primes (e.g., Mok and Morris, 2009). Similarly, the effects of cognitive representation are most likely to be observed with outcomes more tied to identity structure (Miramontez et al., 2008; Saad et al., 2013; Zhang et al., 2014). Future research could tease apart those possibilities to better understand the differential effects of the two components of identity integration.

In addition to the magnitude and consistency of the predicted effects, a main limitation of this research is the correlational nature of our studies. We could not show unambiguously that bicultural identity integration causes less endorsement of dialectical self-beliefs, which in turn leads to higher self-consistency. A viable alternative model is that identity integration and self-consistency are related because they are both *consequences* of holding dialectical self-views. There is some evidence suggestive of causality flowing from dialecticism to identity-related constructs. Sanchez et al. (2009) found among multiracial adults that relative to other mixed-race individuals, Asian/White individuals altered their racial identities more across contexts. Such group difference was possibly the consequence of exposure to dialecticism among Asian/White biracial individuals. This interpretation brings up the influence a bicultural person’s heritage culture could have on the need to integrate different cultural identities. The reverse directionality may be particularly true of East Asian biculturals as their dialectical heritage might set them up for being more tolerant of unintegrated cultural identities in the first place. Thus, there may well be a reciprocal relation between identity integration and dialectical beliefs. To investigate this, researchers could employ a longitudinal panel design to test the respective strengths of identity integration predicting dialectical self-beliefs vs. dialectical self-beliefs predicting identity integration in different bicultural groups.

### Implications

A novel contribution of this research lies in shedding new light on self-consistency that has primarily been studied in the context of cultural comparisons along national and ethnic lines. It is the first to demonstrate the connection of self-consistency with a process biculturals use to negotiate their cultural identities, thus filling in an important knowledge gap. To be specific, the present findings exemplify the value of exploring unique psychological products of being bicultural (Tadmor et al., 2012; Saad et al., 2013; West et al., under review). Bicultural experience may give rise to psychological characteristics that go beyond what may be predicted by each source culture in isolation. In our case, H1 and H2 represent novel predictions derived from the view that regards self-consistency as being shaped by the process of integrating identities associated with multiple cultures; instead, H3 operated from the prevailing assumption that self-consistency is largely influenced by one’s cultural heritage alone. We showed that self-consistency is grounded in the dual-culture maintenance challenge shared among various bicultural groups in addition to heritage cultures that vary from one to another. As a matter of fact, without an explicitly bicultural perspective, it would not have been evident that there may be more than one cultural source that shapes biculturals’ level of self-consistency. Therefore, to predict a bicultural person’s level of self-consistency, we would first need to know his or her cultural heritage (i.e., whether dialectical or not) that serves as a baseline. His or her bicultural experience (i.e., identity integration) would then help fine-tune how far and in which direction the individual level of self-consistency deviates from the baseline.

More generally, in studying the experience of being bicultural, researchers need to be more mindful of connecting the psychological phenomenon of interest with specific aspects of the cultural contexts surrounding biculturals. In addition to heritage cultural norms and beliefs, biculturals could be unique in term of their identity negotiation processes, social status, obstacles to full inclusion, cultural adaptation, and so forth. In the case of Asian Canadians, for instance, whether it is their higher levels of social anxiety (Hsu et al., 2012), experience of lower relational mobility (Zhang and Li, 2014), or compensatory conformity to the European Canadian norm (Tafarodi et al., 2002), there are likely more than one socio-cultural factor that account for those psychological characteristics. To be sure, depending on the form of culture, being bicultural could entail juggling any two or multiple important social identities, such as being a mother and being a career woman with contrastive prescriptions for what it means to succeed (Hodges and Park, 2013; Meeussen et al., 2016). Future research could examine if a similar process to what was found here operates at the intersection of social identities that are receiving increasing attention as social change progresses.

How do the findings relate to the work on dialectical thinking and biculturalism? We think the present research furthers our understanding of individual differences and movement in dialectical thinking in an ever-changing world. Like much cultural psychological research, research on naïve dialecticism construes individual differences in dialectical thinking as largely product of one’s ethnic or national culture. However, our findings suggest that one need not to be explicitly steeped into East Asian philosophies to become dialectical: a dialectical view could arise out of recognizing contradiction and uncertainty in one’s life. For some biculturals (e.g., Latino Canadians or Americans), the complexity of traversing two cultural worlds that are not perfectly aligned with each other could gradually prime them to think and feel in a more dialectical manner, even if neither culture promotes dialecticism. As for biculturalism research, the present research underscores the need to further investigate the psychological and socio-cognitive consequences of bicultural identity integration (e.g., Cheng et al., 2008; Saad et al., 2013) and to differentiate the cognitive from the affective dimension in their downstream effects (e.g., Miramontez et al., 2008; Mok and Morris, 2009).

Finally, results from this research have implications for understanding biculturals’ psychological adjustment. Overall, previous literature shows a more integrated bicultural identity to contribute to greater well-being, but the mechanisms are not well-understood (Downie et al., 2006; David et al., 2009; Huynh et al., 2011). Given that one or both dimensions of identity integration was found to relate to dialectical self-beliefs (either the overall DSS or at least one subscale) in the studies reported here, one specific reason why a lack of identity integration reduces well-being may be that it encourages the tendency to accept both positive and negative sides of oneself and the surrounding social world. Holding dialectical beliefs is indeed somewhat detrimental to well-being, even in East Asian cultures (Spencer-Rodgers et al., 2010). Further supporting the role of dialecticism, there is some evidence that dialectical self-beliefs mediated the effect of identity integration on well-being, at least among biculturals in Hong Kong and China (Chen et al., 2013). Coincidentally, because we used Rosenberg’s self-esteem scale to assess self-evaluative ambiguity in Study 4, global self-esteem scores could be computed to index well-being. Unsurprisingly, global self-esteem was correlated negatively with overall DSS (*r* = -0.52, *p* < 0.001) and specifically harmony (*r* = -0.26, *p* < 0.001). A mediation test confirmed that the effect of harmony on global self-esteem occurred via DSS: 95% CI [0.09, 0.34]. Therefore, dialectical self-beliefs may be one mechanism through which a lack of identity integration comes to negatively impact biculturals’ psychological well-being.

## Conclusion

It is a bicultural mind a consistent mind? The answer appears to depend on two factors. On the one hand, cultural content clearly matters. Because a dialectical cultural tradition attaches less importance to the need to maintain consistency, a bicultural who is enculturated to that tradition tends to develop a less consistent self-concept than one who is enculturated to a non-dialectical tradition. On the other hand, our research highlights the process of integrating more than one culture within the self, irrespective of cultural content. A bicultural mind is a consistent mind when one manages to weave disparate cultural identities into a coherent whole. Ultimately, what unites these two factors is that dialectical beliefs seem to be the chief mechanism by which both come to shape self-consistency in biculturals.

## Author Contributions

RZ and KN designed the studies. RZ, SS, and RL collected the data. RZ performed the data analysis. RZ drafted the manuscript and KN and RL provided critical feedback. The authors would like to acknowledge the Dickinson College Research & Development Committee and the Social Sciences and Humanities Research Council of Canada for their support of this publication.

## Conflict of Interest Statement

The authors declare that the research was conducted in the absence of any commercial or financial relationships that could be construed as a potential conflict of interest.

## Footnotes

1 In all studies, we did not find any systematic generational difference in the patterns of the results. Given the relatively small sample sizes of each generation in some of the studies, however, we cannot rule out this possibility, which future research could examine (e.g., Zhang and Noels, 2013).

2 We also included the Bicultural Self-Efficacy Scale (BSES; David et al., 2009), which assesses the perceived competence for biculturals to function effectively and maintain meaningful relationships in both cultures. The original scale contains six subscales and we administered four of them: social groundedness (SG; α = 0.91) which refers to the extent to which a bicultural person’s social networks consist of people from both cultures, positive attitudes (PA; α = 0.89) which refers to the extent to which a bicultural person holds both cultures in high regard, role repertoire (RR; α = 0.64) which reflects a bicultural person’s range of culturally appropriate skillsets, and bicultural beliefs (BB; α = 0.80) which are the expectation that biculturals can function effectively in both cultures. The four subscales, particularly SG and PA, were also found to relate positively to global consistency in self-evaluations. Furthermore, DSS mediated most of those effects. Although bicultural self-efficacy is distinct from bicultural identity integration (David et al., 2009; in this study, *r*s between BII and BSES subscales ranging from 0.24 to 0.45), findings regarding BSES provide convergent evidence for our general argument that bicultural experience influences self-consistency. Detailed results can be obtained from the first author.
